# Hepatopulmonary syndrome associated with long-term use of ado-trastuzumab emtansine (T-DM1) for treatment of HER2-positive metastatic breast cancer - a case report and series

**DOI:** 10.3389/fonc.2024.1434492

**Published:** 2024-10-21

**Authors:** Ciara C. O’Sullivan, Alexandra S. Higgins, Adham K. Alkurashi, Vaibhav Ahluwalia, Jodi L. Taraba, Paul M. McKie, Patrick S. Kamath, Vivek N. Iyer, Tufia C. Haddad

**Affiliations:** ^1^ Department of Oncology, Mayo Clinic, Rochester, MN, United States; ^2^ Division of Pulmonary and Critical Care Medicine, Mayo Clinic, Rochester, MN, United States; ^3^ Department of Pharmacy, Mayo Clinic, Rochester, MN, United States; ^4^ Department of Cardiology, Mayo Clinic, Rochester MN, United States; ^5^ Division of Gastroenterology and Hepatology, Mayo Clinic, Rochester, MN, United States

**Keywords:** antibody-drug conjugates (ADCs), ado-trastuzumab emtansine (T-DM1), hepatotoxicity, breast cancer, portal hypertension, hepatopulmonary syndrome (HPS)

## Abstract

**Background:**

The advent of antibody-drug conjugates (ADCs) represents a landmark advance in cancer therapy, permitting targeted delivery of a potent cytotoxic agent to tumor cells with minimal damage to surrounding cells. Although ADCs can induce sustained therapeutic responses in heavily pretreated patients, they can also cause significant toxicity and thus require careful monitoring. The prototype ADC, ado-trastuzumab emtansine (T-DM1) is comprised of a humanized, monoclonal human epidermal growth factor receptor 2 (HER2)-directed antibody, trastuzumab, linked to the cytotoxic agent, DM1, and is used for the treatment of early-stage and advanced HER2-positive breast cancer. Liver toxicities, including transaminitis and nodular regenerative hyperplasia resulting in portal hypertension have been described. We report a case series of four patients who developed hepatopulmonary syndrome (HPS) during treatment with T-DM1. HPS is characterized by hypoxemia, portal hypertension, and intrapulmonary shunting, and it can be associated with severe hypoxic respiratory failure. HPS secondary to noncirrhotic portal hypertension occurring with long-term exposure to T-DM1 has not previously been reported.

**Case series presentation:**

Four patients who received T-DM1 in our institutional cohort (n=230) developed HPS, which can be associated with severe hypoxic respiratory failure. Each patient diagnosed with HPS received >50 doses of T-DM1. Only one patient at diagnosis had resting hypoxia, while the other three patients became hypoxic with exertion only. Discontinuation of T-DM1 led to clinical improvement in hypoxia in three of the four patients. The spectrum of liver injury that occurs with long-term use of T-DM1 remains incompletely defined.

**Conclusions:**

As T-DM1 is approved for use in the management of early-stage operable and advanced breast cancer, awareness of HPS as a potential complication of long-term administration of T-DM1 is necessary. The emergence of dyspnea alone or combined with low oxygen saturation and signs of hypoxemia (clubbing or elevated hemoglobin) should raise clinical suspicion and prompt evaluation for HPS. Cancer care team members should be vigilant regarding the potential for new and serious side effects associated with novel targeted therapies, which may emerge years beyond initial regulatory approval.

## Introduction

Antibody-drug conjugates (ADCs) are a rapidly evolving class of antineoplastic agents which can induce deep, sustained responses in heavily pretreated patients with cancer. The prototype ADC, ado-trastuzumab (T-DM1), was approved by the U.S. Food and Drug Administration (FDA) for treatment of human epidermal growth factor receptor 2 positive (HER2+) metastatic breast cancer (MBC) in 2013 and HER2+ early breast cancer (EBC) in 2019 (1, 2). T-DM1 is comprised of the humanized, monoclonal HER2-directed antibody, trastuzumab, linked to the cytotoxic agent, DM1 (3). It is administered intravenously on day 1 of a 21-day cycle. Liver toxicities are common and can be dose-limiting, to include a transient increase in serum aminotransferase levels, and less commonly, nodular regenerative hyperplasia resulting in portal hypertension (4, 5). Herein we report four patients who developed hepatopulmonary syndrome (HPS) during prolonged T-DM1 treatment. HPS, characterized by hypoxemia, portal hypertension, and intrapulmonary shunting, and manifests clinically as dyspnea, has not previously been reported as a toxicity associated with T-DM1 exposure. This case series serves as a reminder that new and potentially serious adverse effects (AEs) of novel cancer therapies may emerge years after regulatory approval and drug initiation.

Hepatopulmonary syndrome (HPS) is characterized by the presence of hypoxemia due to the occurrence of intrapulmonary vascular dilatations (IPVDs) in the setting of acute or chronic liver disease (with most patients showing signs of cirrhotic or non-cirrhotic portal hypertension). The hypoxemia in HPS occurs due to the intrapulmonary shunting and ventilation-perfusion mismatching caused by the IPVDs. Patients can be asymptomatic or can present with dyspnea, as well as with platypnea and orthodeoxia. The clinical triad required for the diagnosis of HPS include the presence of (1) Hypoxemia, (2) Intrapulmonary shunting (typically confirmed by bubble contrast echocardiography), and (3) Liver disease and/or portal hypertension. Workup should exclude other competing etiologies for hypoxemia and dyspnea in patients with liver disease (6). Portal hypertension may be a result of cirrhosis or non-cirrhotic causes, such as nodular regenerative hyperplasia of the liver associated with drug therapies. HPS is present in 10-30% of patients with end stage liver disease being evaluated for liver transplantation and is associated with significantly higher mortality. The severity of HPS is often unrelated to the severity and/or progression of underlying liver disease (7, 8).

Approximately 15% of breast cancers are HER2+, a molecular subtype traditionally associated with a poor prognosis prior to the advent of the HER2-targeted monoclonal antibody, trastuzumab (9). Given recent rapid therapeutic advances, resulting in approval of multiple HER2-directed therapies, there have been major improvements in oncologic outcomes (10). ADCs in particular have transformed the treatment landscape of HER2+ MBC. ADCs are comprised of a monoclonal antibody linked to biologically active drugs, delivering cytotoxic compounds at the tumor site and limiting systemic exposure and toxicity. They are typically well tolerated, however the potential for AEs (e.g., neutropenia, nausea, diarrhea, left ventricular dysfunction and pneumonitis) requires careful monitoring. Ado-trastuzumab emtansine (T-DM1) is an ADC comprised of trastuzumab covalently linked to the cytotoxic chemotherapeutic agent, DM1, a derivative of the microtubule inhibitor, maytansine (3). T-DM1 retains trastuzumab activity while providing intracellular delivery of DM1 to cells expressing HER2. It is FDA approved for the adjuvant treatment of residual invasive HER2+ EBC following preoperative chemo and HER2-directed therapy (2), and for the treatment of HER2+ MBC after prior trastuzumab plus taxane.(1) In the curative intent, EBC setting, 14 cycles of T-DM1 are recommended; however, for HER2+ MBC, treatment with T-DM1 continues until disease progression or unacceptable AEs. While the median progression-free survival (PFS) of patients receiving T-DM1 as second-line therapy for MBC is 6.8 - 9.6 months (~10-14 cycles), there are exceptional responders who remain on therapy for ≥ 18 months.(1) Trastuzumab deruxtecan (T-DXd) recently replaced T-DM1 as second-line treatment for HER2+ MBC, given its superior PFS when compared with T-DM1 in the DESTINY Breast-03 trial (11). Therefore, T-DM1 has moved to third and later line treatment settings for HER2+ MBC (12).

Clinical development of maytansine in advanced solid tumors was hampered by limited clinical efficacy and dose-limiting toxicities (mainly elevation in serum aminotransferases) (13). Although maytansine monotherapy was too toxic, when its analog, DM, was linked to trastuzumab, the resulting ADC, T-DM1, offered a targeted and safer delivery system for the potent chemotherapy to treat HER2+ breast cancer. Preclinical studies of T-DM1 noted its association with elevations in aminotransferase levels, and histopathologic changes in the liver, including hepatocellular and biliary necrosis (14). Although T-DM1 is overall well tolerated, liver toxicities are well described, including dose-limiting, transient increases in aminotransferase levels, and less commonly, nodular regenerative hyperplasia resulting in portal hypertension (4, 5). HPS secondary to noncirrhotic portal hypertension in the context of T-DM1 exposure has not previously been reported. Herein, we present our single institution case series of four patients with HER2+ MBC who developed HPS while receiving T-DM1.

The aim of this report is to characterize the clinical course of HPS and establish awareness of this complication in the setting of prolonged T-DM1 administration, as early detection could potentially prevent irreversible hypoxic respiratory failure.

## Case series presentation

### Methods

This case series included a review of all patients who received at least one dose of T-DM1 in the adjuvant or metastatic setting for treatment of HER2+ breast cancer between January 2013 and October 2020 at the Mayo Clinic. Institutional tools that leverage natural language processing to search the electronic health record (EHR) were utilized to identify patients who received T-DM1 at any Mayo Clinic site. The research was classified as IRB exempt. All patients had provided signed authorization for use of their personal health information for retrospective research.

Duration of exposure to T-DM1 and clinical, laboratory, and imaging data were abstracted from the EHR. This included all available liver function tests one year prior to, at the start of, during, and at completion of T-DM1, as well as oxygen saturation levels and imaging performed within one month of starting and discontinuing T-DM1. Abdominal imaging was reviewed for the presence of hepatic nodularity and markers of portal hypertension, such as portosystemic collaterals, ascites, and splenomegaly. Thoracic imaging was reviewed for signs suggestive of right heart failure (cardiomegaly, lung parenchymal disease). AE reporting utilized the Common Terminology Criteria for Adverse Events v5.0 (15).

Evidence for HPS was recorded, to include all of the following criteria: (1) hypoxemia (Pa02 < 80 mmHg), (2) portal hypertension (any or all of the following: esophageal and/or gastric varices, high serum-ascitic fluid albumin gradient, ascites, portosystemic collaterals, and splenomegaly), and (3) IPVD related intrapulmonary shunting confirmed by contrast echocardiogram or Tc-99m macroaggregated albumin (MAA) lung perfusion scan (6). Normal shunting to the brain with Tc MAA (macro-aggregated albumin) scan is <6%. The cut off for a diagnosis of HPS was > 6% shunting to brain. For those meeting a HPS diagnosis, the clinical signs and symptoms, laboratory findings, and diagnostic medical imaging were independently reviewed by a hepatologist [author PSK] to confirm the absence of preexisting liver disease. Clinical outcomes for the cohort of patients who developed HPS were followed by EHR review through November 2021.

## Results

Two hundred thirty women with HER2+ breast cancer treated with T-DM1 in the adjuvant (stage 1-3) (n=78) or metastatic setting (n=152) were identified. Median age was 57 years (range 27-92), and median prior lines of systemic therapy received prior to T-DM1 was 1.0 (range 0-8). The median number of T-DM1 cycles administered was 10 (range 1-106). During T-DM1 treatment, ≥1 episode of elevated bilirubin and/or liver aminotransferases (AST or ALT) were experienced by 13.5% (n=31) and 80% (n=184) of patients, respectively.

### T-DM1-associated HPS cohort

Four patients (1.7%) met all 3 criteria for a diagnosis of HPS while receiving T-DM1. Case summaries are provided in **Table 1**. Three patients were non-Hispanic, white and one patient was Asian. All four had MBC, involving lymph nodes (n=4), bone (n=3), and/or liver (focal metastases; n=2). None of the patients had pleural or pulmonary metastases. Median age at HPS diagnosis was 43 (range 35 – 53 years). The median number of lines of systemic therapy received prior to T-DM1 was 1.5 (range 0 – 7), and the median number of cycles of T-DM1 administered was 68.5 (range 51 – 90 cycles which equates to approximately 35 – 62 months).

**Table 1:** Summary of patients exposed to T-DM1 who developed hepatopulmonary syndrome.

Case	Age at HPS onset (years)	Sites of MBC disease	Prior lines of therapy (#)	Cycles of T-DM1 (#)	Signs or symptoms at onset	Oxygen requirement	Non-contrast echo findings	Transaminitis; hyper-bilirubinemia	Evidence of portal hypertension	Hgb prior to T-DM1 (g/dL)	Hgb post- T-DM1 (g/dL)	Tc-99m MAA Lung perfusion study	Contrast echo	Current Status
**1**	53	LN, Bone	0	90	DOE, clubbing, hypoxemia	Yes	Left atrial enlargement (volume index 62 ml/m^2^)	No	Portovenous collaterals, esophageal varices, splenomegaly	12.9	10.7	13.5%	Small shunt	Dead
**2**	35	LN, bone, liver	7	51	DOE	Yes	Left atrial enlargement (volume index 54 ml/m^2^)	Grade 1; Grade 2	Splenomegaly, esophageal-gastric varices, telangiectasias	Not known	11.9	24%	Large shunt	Unknown
**3**	38	LN, bone	2	51	High bilirubin, DOE	Yes	Borderline left atrial enlargement	Grade 1; Grade 2	Splenomegaly, esophageal-gastric varices, telangiectasias	11.7	16.2	17.8%	Moderate shunt	Alive
**4**	48	LN, liver	1	86	DOE	No	Left atrial enlargement (volume index 49 ml/m^2^)	Grade 1; Grade 2	Portosystemic varices, splenomegaly, telangiectasias	12.5	16.1	7.9%	Large shunt	Alive

DOE, dyspnea on exertion; g/dl, grams per deciliter; Hgb, hemoglobin; HPS, hepatopulmonary syndrome; LN, lymph nodes; N/A, not applicable; MBC, metastatic breast cancer; ml/m^2^, milliliter per square meter; T-DM1, trastuzumab emtansine; UNK, unknown.

### Clinical presentation and diagnosis

Notably, all patients presented with dyspnea on exertion and were found to have arterial hypoxemia. Portal hypertension, as determined by the presence of portosystemic collaterals and splenomegaly on CT abdomen, was present. Intrapulmonary shunt was confirmed by contrast echocardiogram or lung perfusion scan. Three patients had cutaneous spider telangiectasias, as well as grade 1-2 elevations in serum transaminases and bilirubin. Three patients required supplemental oxygen, and elevation of hemoglobin was noted in two patients.

### Clinical outcomes

Patient 1 had a diagnosis of intrapulmonary shunt while on T-DM1. She died two months after stopping T-DM1 due to disease progression in her lymph nodes and bones. Her cause of death was unknown as she was lost to follow-up, however HPS secondary to T-DM1 was suspected (diagnosed post-mortem based on chart review). The other three patients experienced stability or clinical improvement from the HPS standpoint after discontinuing T-DM1. Patient 2 remained off cancer-directed treatment for several months, but eventually her metastatic disease progressed. She subsequently received trastuzumab, pertuzumab plus chemotherapy intermittently for an additional 28 months (as of last correspondence with her local oncologist). During this time, she did not experience worsening of her hypoxemic respiratory failure. Two years after patient 3 discontinued T-DM1, her bilirubin normalized, cutaneous spider telangiectasias regressed, and hypoxemia improved (arterial blood gas PaO2 of 65 mmHg compared with baseline 59 mmHg and Sp02 92% with exertion compared with baseline 78% with exertion). An echocardiogram performed 4 years after T-DM1 discontinuation showed a stable moderate intrapulmonary shunt with no other significant findings. At her last clinical follow up, 5.5 years after T-DM1 discontinuation, HPS had resolved, and CT scans showed no active metastases. Remarkably, she had not required cancer-directed systemic therapy since T-DM1 discontinuation. Patient 4 presented with grade 1 dyspnea on exertion and isolated left atrial enlargement on echocardiogram. An oxygen consumption study demonstrated exercise-induced hypoxia. This prompted an echocardiogram shunt study that confirmed the presence of a large intrapulmonary shunt. She had a complete radiologic response to T-DM1 at treatment discontinuation, which was sustained at 3 years without any cancer-directed therapy. Due to echocardiographic evidence of increasing pulmonary arterial pressures 3 years after treatment discontinuation, she underwent hemodynamic catheterization confirming moderate pulmonary hypertension (mean 38 mm Hg) with normal right atrial and left wedge pressures. She commenced macitentan (an endothelin receptor antagonist used for the treatment of pulmonary arterial hypertension) and continues to report grade 1 dyspnea.

## Conclusion

We report HPS as a newly described and potentially life-threatening complication that can occur in the setting of prolonged T-DM1 exposure. The mechanism of T-DM1 induced hepatotoxicity has not been fully defined, but may be due to endothelial cells and vasculature injury, potentially by the microtubule inhibitor conjugate (16). Factors complicating recognition of T-DM1 associated HPS include lack of knowledge regarding the condition, a low index of suspicion, and the fact that the diagnosis may be made after disease progression on T-DM1. Choi et al. noted higher incidences of noncirrhotic portal hypertension, splenomegaly, gastroesophageal varix formation, and spontaneous portosystemic shunting in patients treated with T-DM1 compared with those treated with the HER2-targeted tyrosine kinase inhibitor, lapatinib, in combination with capecitabine (a 5-Flourouracil prodrug) (17). One other real-world, institutional experience of all patients who received T-DM1 for a median of 11 cycles (range 1 – 67) noted a 19.5% rate of grade ≥3 hepatic dysfunction, which is higher than reported in prior trials (18). The findings were attributed to prior taxane chemotherapy and the presence of liver metastases in over half of the patients studied. In a separate analysis prospectively evaluating the safety of long-term T-DM1 use, of 115 patients who received a median 18 cycles of therapy (range 12 -59), no increase in incremental toxicity (specifically hepatotoxicity) was noted in patients with liver metastases (19). With regards to the four patients who developed HPS in our case series, the median cycles of therapy received was 68.5 (range 51 – 90), therefore this treatment complication may be associated with a longer than average duration of T-DM1 therapy.

In all four patients, the clinical manifestation of HPS was dyspnea on exertion. Therefore, the emergence of dyspnea alone or combined with low oxygen saturation or signs of hypoxemia (clubbing or elevated hemoglobin) should raise clinical suspicion and prompt evaluation for a potential diagnosis of HPS. Patients receiving curative intent, HER2-directed therapy, are commonly monitored with serial echocardiograms every 3 months as trastuzumab can cause reversible cardiomyopathy and a drop in left ventricular ejection fraction (20). Cardiac surveillance is not standardized in the metastatic disease setting or with other HER2-directed agents, and it should be performed based on a risk-benefit approach (21). The emergence of dyspnea on exertion while receiving HER2-directed therapy is likely to prompt a transthoracic echocardiogram amongst other testing. Findings indicative of HPS on non-contrast echocardiogram may be subtle and include left atrial enlargement (22). As the latter is non-specific, it is important to perform an agitated saline contrast echocardiogram to assess for intrapulmonary shunting when there is clinical suspicion for HPS (23). If not diagnosed early in the disease trajectory and T-DM1 is continued, HPS may result in chronic, severe hypoxic respiratory failure, pulmonary hypertension, and right ventricular dysfunction. Therefore, it is imperative that clinicians are aware of the constellation of symptoms, signs, and imaging findings indicative of this condition.

With regards to the clinical implications of our findings, as the median overall survival (OS) for patients with HER2+ MBC approaches 5 years (10), many patients receive several lines of HER2-directed therapy, and T-DM1 will continue to be commonly prescribed for disease management. The efficacy of T-DM1 after treatment with T-DXd is not well-described (24); however, given different chemotherapeutic payloads, it is feasible there will be clinically meaningful responses to T-DM1 (25). The incidence of exceptional responders who have prolonged exposure to T-DM1 after T-DXd remains unknown. Nevertheless, our findings remain relevant in settings where T-DXd is not readily available or is contraindicated, and for those patients who wish to receive an ADC without risk for alopecia. Further, as the pathophysiology of T-DM1-induced HPS remain unclear, it is feasible that this complication could occur with long- term exposure to other ADCs, particularly microtubule-based compounds. Therefore, it remains clinically relevant to raise awareness of HPS as an uncommon but potentially life-threatening complication in this setting, especially as there are > 100 ADCs in clinical development for the treatment of various liquid and solid malignancies (26, 27).

Further data are needed to better characterize the spectrum of liver injury that occurs with long-term T-DM1 use. In the interim, we propose a focused workup for patients on prolonged T-DM1 with a suspected diagnosis of HPS (**Table 2**), and we provide guidelines for managing same (**Table 3**). From a postmarketing surveillance standpoint, as phase IV trials may have smaller sample sizes, additional education on and participation by healthcare providers in spontaneous reporting processes is critical to avoid gaps in clinical safety data (28). As novel targeted therapies have improved OS in many patients and may be continued for long durations, it is critical to highlight the need for continuous assessment of long-term tolerability and monitoring for potentially harmful late effects of treatment (29). This is especially true regarding rare AEs such as HPS, which are not identified in clinical development and may only be reported when the drug is prescribed to large volumes of patients in routine clinical practice. Although this review was developed to serve as a resource for oncologists, advanced practice providers, nurses, and pharmacists treating patients with prolonged T-DM1, the overarching message is that all care team members must be vigilant, as new and potentially serious side-effects associated with established cancer-directed therapies may emerge well beyond initial approval.

**Table 2:** Clinical assessment for symptoms and signs of T-DM1 associated hepatopulmonary syndrome.

**1. Clinical evaluation** a. Inquire about dyspnea at rest and/or exertion. b. Assess resting room air with pulse oximeter. If Sp02 < 96%, then pursue further workup for evaluation of hypoxemia/shunting. c. Assess for signs of liver disease (elevated bilirubin, transaminitis, spider naevi, splenomegaly, ascites etc.). **2. Laboratory/diagnostic tests:** a. ABG evaluation: can confirm A-a gradient ≥ 15 mmHg and evaluate the severity of hypoxemia based on Pa02 level. b. Contrast enhanced (bubble) echocardiography: most sensitive test to evaluate for presence of intrapulmonary shunting due to IPVDs. c. CT Chest: can exclude competing etiologies for hypoxemia such as pleural effusions, interstitial lung disease etc. d. Tc-99m MAA lung perfusion study: can quantitate degree of intrapulmonary shunting (normal < 6%). e. MRI abdomen with elastography or FibroScan, +/- liver biopsy +/- upper endoscopy: can confirm presence of liver disease, cirrhosis, and/or portal hypertension. f. Lung function testing (DLCO): to determine if low diffusing capacity may be present

Sp02, oxygen saturation; ABG, arterial blood gas; IPVDs, intrapulmonary vascular dilatations; MAA, macroaggregated albumin; PaO2, the partial pressure of oxygen in the arterial blood; DLCO, diffusing capacity of the lungs for carbon monoxide.

**Table 3:** Management of T-DM1 associated hepatopulmonary syndrome.

1. Oncology: Discontinue T-DM1. Of note, it is reasonable to cautiously continue T-DM1 in selected patients with mild HPS. If disease stable, or no evidence of disease, consider observing off systemic therapy. Otherwise, prescribe alternative HER2-targeted therapy with no or minimal risk of liver or pulmonary toxicity. 2. Cardio-Pulmonary: Prescribe supplemental oxygen, as needed. Exclude other causes of hypoxemia. 3. Hepatology: No specific measures other than treat cause. Hold T-DM1 and minimize exposure to other hepatotoxic agents, if possible.

## Data Availability

The original contributions presented in the study are included in the article/supplementary material. Further inquiries can be directed to the corresponding author.
